# “Non-thermal” ablation using focused ultrasound and an ultrasound contrast agent

**DOI:** 10.1186/2050-5736-3-S1-O9

**Published:** 2015-06-30

**Authors:** Nathan McDannold

**Affiliations:** 1 Brigham & Women’s Hospital/Harvard Medical School, Boston, MA, United States

## Background/introduction

The combination of focused ultrasound and an intravenously-injected microbubble ultrasound contrast agent offers the ability to reduce the power needed to ablate tissue, which is particularly important for targets in the brain since it can eliminate skull heating. When applied at a low duty cycle so that bulk tissue heating does not occur, the mechanical effects induced by inertial cavitation result in ablation via the destruction of blood vessels. This alternative method for tissue ablation may have advantages for the treatment of brain tumors.

## Methods

Primates at our institution that evaluated the bioeffects induced during contrast-enhanced ultrasound ablation of targets in the brain. These studies applied burst sonications slightly above the inertial cavitation threshold at a low ultrasound frequency (220-525 kHz) and a 1% duty cycle over several minutes. Each sonication was applied after a bolus injection of ultrasound contrast agent.

## Results and conclusions

Such contrast-enhanced sonications produce vascular damage, which leads to a localized infarct and tissue necrosis. The necrotic tissue is removed quickly compared to thermal ablation. When volumes are ablated, a cavity reminiscent of surgical resection is evident in 2-3 weeks. White matter tracts, which have a low vascular density, appear to be somewhat resistant to this type of ablation. No evidence of thermal damage is evident.

The combination of focused ultrasound and a microbubble agent can enable ablation of tumors or other targets in the brain over a wider envelope than is currently possible with thermal ablation. The resulting bioeffects may offer advantages for tumors. The relatively quick removal of tissue may be helpful to reduce adverse effects resulting from tumor bulk effects. The resistance of large white matter tracts to these sonications may enable ablation of tumors directly adjacent to cranial nerves which is a current challenge for tumors located near the skull base. However, significantly more work is needed to establish the viability of this technique in tumors and to understand how the method can be applied safely. Major issues needing resolution will be a topic for discussion.

